# Inositol Pyrophosphates: Signaling Molecules with Pleiotropic Actions in Mammals

**DOI:** 10.3390/molecules25092208

**Published:** 2020-05-08

**Authors:** Seulgi Lee, Min-Gyu Kim, Hyoungjoon Ahn, Seyun Kim

**Affiliations:** 1 Department of Biological Sciences, Korea Advanced Institute of Science and Technology (KAIST), Daejeon 34141, Korea; seulgi1018@kaist.ac.kr (S.L.); kimmg711@kaist.ac.kr (M.-G.K.); toddhwhd@kaist.ac.kr (H.A.); 2 KAIST Institute for the BioCentury, KAIST, Daejeon 34141, Korea

**Keywords:** inositol pyrophosphate, IP6K, PPIP5K, cell signaling, physiologic functions, signaling molecules

## Abstract

Inositol pyrophosphates (PP-IPs) such as 5-diphosphoinositol pentakisphosphate (5-IP7) are inositol metabolites containing high-energy phosphoanhydride bonds. Biosynthesis of PP-IPs is mediated by IP6 kinases (IP6Ks) and PPIP5 kinases (PPIP5Ks), which transfer phosphate to inositol hexakisphosphate (IP6). Pleiotropic actions of PP-IPs are involved in many key biological processes, including growth, vesicular remodeling, and energy homeostasis. PP-IPs function to regulate their target proteins through allosteric interactions or protein pyrophosphorylation. This review summarizes the current understanding of how PP-IPs control mammalian cellular signaling networks in physiology and disease.

## 1. Introduction

Myo-inositol (inositol), a six-carbon glucose isomer with one axial and five equatorial hydroxyl groups, is a key nutrient in the human [1,2]. In mammals, inositol can be produced from the isomerization of glucose-6-phosphate by inositol 3-phosphate synthase to inositol 3-phosphate, which is dephosphorylated by inositol monophosphatase 1 to yield free myo-inositol. In plants, inositol hexakisphosphate (IP6) is known as phytic acid, which is utilized for phosphorus storage [3]. When the levels of inositol are insufficiently maintained in the body, serious medical complications, including anxiety disorders, diabetes, and hypercholesterolemia, may occur [1,2,4].

**Figure 1:**
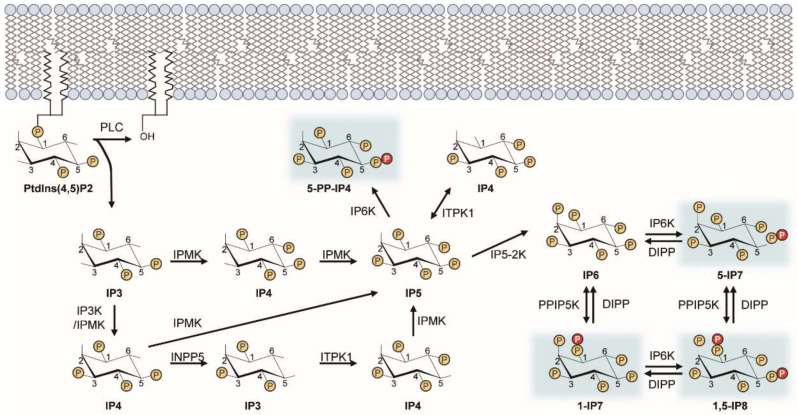
Inositol pyrophosphate biosynthetic pathway. This diagram illustrates the metabolic routes of inositol poly- and pyro-phosphate synthesis in mammalian cells. Sequential phosphorylation of IP3 by IPMK and other IP kinases (e.g., IP3-kinases, IP5-2K) yields IP6. Pyrophosphorylation of IP6 by IP6Ks and PPIP5Ks finally leads to the production of PP-IPs (e.g., 5-IP7). PP-IPs are highlighted in blue. ITPK, inositol trisphosphate 3-kinase; IPMK, inositol polyphosphate multi-kinase; INPP5, inositol polyphosphate 5-phosphatase; IP5-2K, inositol pentakisphosphate 2-kinase.

In mammalian cells, inositol is primarily found as a structural component of phosphatidylinositols that helps maintain cellular membranes [1]. The pioneering discovery of phospholipase C in the late 1980s revealed the importance of inositol as an important bioactive secondary messenger of cellular signaling [5]. Bioactive inositols, inositol polyphosphates (IPs) such as inositol 1,4,5-trisphosphate (IP3), contain more than one phosphate. An example of bioactive inositol activity is IP3-mediated cytosolic calcium release [6,7]. Phospholipase C is activated by growth factor stimulation and cleaves the phosphatidylinositol 4,5-bisphosphate (PIP2), producing IP3 and the lipid-anchored diacylglycerol. IP3 released from the membrane to the cytosol subsequently binds to and opens the IP3 receptor, an IP3-gated calcium channel that regulates cytosolic calcium levels. IP3 becomes further metabolized into higher phosphorylated IPs, e.g., IP4, IP5, IP6, and IP7, through specific inositol phosphate kinases [2,7,8,9] (Figure 1). In this review, we focus on the recent progress in the biological actions of inositol pyrophosphates in mammals.

## 2. Biosynthesis of Inositol Pyrophosphates

Among many IP species, pyrophosphorylated-IPs (PP-IPs) exhibit a unique structural feature in that they contain highly energetic phosphoanhydride bonds (pyrophosphates) at specific positions [10,11]. Their biosynthesis is catalyzed by two groups of IP kinases, the IP6 kinases (IP6Ks) [12,13] and the PPIP5 kinases (PPIP5Ks) [14,15], which phosphorylate IP6 and IP7. In mammals, three IP6Ks (IP6K1, IP6K2, and IP6K3) phosphorylate the 5 position of IP6 to form 5-PP-IP5 (5-IP7) [12,13]. Two PPIP5Ks (PPIP5K1 and PIPP5K2) can transfer a phosphate to the 1 position of IP6 to produce 1-PP-IP5 (simply designated as 1-IP7). IP8 with two pyrophosphates at the 5 and 1 positions is synthesized when IP6 is fully phosphorylated by both IP6Ks and PPIP5Ks [14,15,16,17,18] (Figure 1). The major source of IP8 synthesis proceeds through 5-IP7, not 1-IP7. The enzyme diphosphoinositol polyphosphate phosphohydrolase (DIPP) is responsible for dephosphorylating PP-IPs. Compared to 5-IP7, 1-IP7 appears to be a better substrate for DIPP [19], which can result in higher cellular levels of 5-IP7 than 1-IP7 [20]. Dominant phosphorylation of IP6 by IP6Ks at the 5 position over PPIP5K-dependent phosphorylation at the IP6’s 1 position may also explain the high level of 5-IP7, as well as the rapid conversion of 1-IP7 to IP8.

At steady state, the concentration of IP7 in cultured mammal cells varies between 0.5 and 5 μM [16,21,22]. The level of IP8 is much less than 10%‒20% of the concentration of 5-IP7 [20]. The cellular levels of PP-IPs dynamically fluctuate. In cultured primary hepatocytes, IP7 turns over every 4 min, whereas IP6 turns over every 40 min [23]. Importantly, the metabolic flux of PP-IP can be influenced by cellular conditions. In ovarian cancer cells, staurosporine treatment results in a five-fold increase in IP7 [24]. In addition, IP7 levels are shown to change in a cell-cycle-dependent fashion in tumor cell lines [22]. In serum-starved mouse embryonic fibroblasts, IP7 is depleted [25]. A two-fold increase of IP7 was reported from insulin-stimulated mouse primary hepatocytes [25]. In addition to IP7, cellular conditions such as osmolarity [14], temperature [26], and phosphate levels [27] are also known to increase IP8 levels. Because of their high-energy phosphate bonds and rapid cellular turnover in response to cellular stimuli, PP-IPs have been viewed as ‘mammalian cell signals’, ‘regulators of cell homeostasis’, and ‘metabolic messengers’ [9,11,28,29,30].

## 3. The Modes of Action of the Inositol Pyrophosphates

It is challenging to characterize the mechanistic basis underlying the cellular and physiological changes regulated by PP-IPs. Similar to signaling molecules, PP-IPs modulate specific target proteins to control cellular signaling events via different molecular interactions [9,11,31]. Non-covalent interactions between these metabolites and proteins occur allosterically or stably. In common with other major signaling molecules, such as cyclic AMP [32], allosteric interactions between PP-IPs and their protein targets are the representative mode of PP-IP action [11,31].

**Figure 2:**
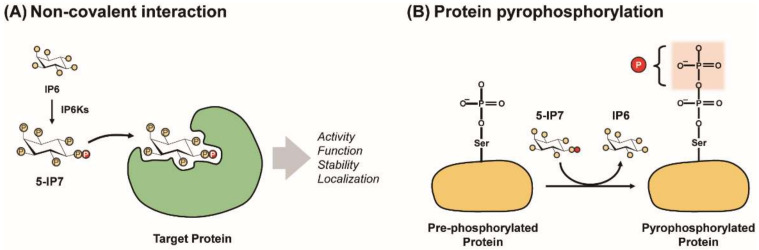
Modes of PP-IPs’ signaling action. PP-IPs can modulate characteristics of target proteins (e.g., activity, function, localization, and stability) via either the allosteric PP-IP−protein interaction (**A**) or protein pyrophosphorylation (**B**).

As metals act as protein structural cofactors in cells, some metabolites can form stable interactions with proteins to promote and sustain proper protein folding and stability. However, no stable interaction partner for PP-IPs has been elucidated. An additional metabolite signaling action is the covalent modification of proteins; metabolites serve as substrates for protein modification (e.g., ATP and succinyl-CoA) and can induce chemical changes on proteins, to regulate functioning or biochemical fates, such as protein stability and subcellular localization. Furthermore, PP-IPs are known as the source for protein pyrophosphorylation [11,31,33,34] (Figure 2).

### 3.1. Allosteric Binding of PP-IPs with Proteins

Target proteins for 5-IP7, 1-IP7, and IP8 are regulated by allosteric binding of their respective PP-IPs, similar to the discovery that the IP3-gated Ca^2+^ channel is an IP3 receptor [35]. For example, Akt recruitment to phosphatidylinositol 3,4,5-trisphosphates (PIP3) in the plasma membrane is required for full activation to occur. The competitive inhibitor, 5-IP7, binds to the PH domain on Akt, which blocks the Akt-PIP3 interaction [25]. Furthermore, 5-IP7 can bind allosterically to casein kinase 2 (CK) to promote its phosphorylation of TTT (Tel2, Tti1, and Tti2) in response to DNA damage, which stabilizes and enhances DNA-dependent protein kinase catalytic subunit (DNA PKcs)/ATM kinase/p53-mediated apoptosis [36]. Another 5-IP7 binding target is synaptotagmin1 (Syt1), which is a calcium sensor that mediates synaptic vesicle exocytosis in the presynaptic terminal. High-affinity interactions between 5-IP7 and Syt1 can lock Syt1 into an inactive state, which suppresses synaptic vesicle exocytosis and neurotransmitter release [37]. To date, interferon response factor 3 (IRF3) has been proposed as a 1-IP7-specific protein target in type I interferon immunity [38]. In addition, another recent finding demonstrated that IP8 specifically binds to the N-terminus of XPR1 (Xenotropic and polytrophic retrovirus receptor 1) with high affinity, leading to XPR1-mediated phosphate export [39].

### 3.2. Protein Phosphorylation by PP-IPs

Another mode of PP-IP action is the pyrophosphorylation of target proteins. Similar to adenosine trisphosphate (ATP), PP-IPs can act as high-energy phosphate donors due to the energy in the phosphoanhydride bonds that powers the attachment of their β-phosphates to the protein target [33,34]. Indeed, PP-IPs can non-enzymatically transfer their β-phosphates to serine residues that have been primed by phosphorylation by CK2. In this manner, CK2 provides the phosphoprotein substrates for PP-IP-mediated pyrophosphorylation because acidic amino acids surrounding the targeted phospho-Ser correspond to the consensus motif for CK2 [33,34,40]. PP-IPs’ protein pyrophosphorylation appears to be involved in many biological events, such as glycolysis [41], HIV particle release [42], and dynein-mediated trafficking [43]. Mass spectrometric validation and functional studies on this unique post-translational modification will further expand our appreciation of PP-IPs’ signaling actions.

## 4. The Biological Actions of PP-IPs

Since the establishment of IP3′s central role in the control of cellular calcium homeostasis [6], PP-IPs have been assumed to mediate their own selective signaling and associated biological functions [9,10,11,30]. In efforts to investigate the roles of PP-IPs in vitro and in vivo, groups of researchers have modulated cellular levels of PP-IPs by overexpression or deletion of enzymes responsible for PP-IP biosynthesis [9]. Since IP6Ks were discovered in 1999 [12,13], earlier than PPIP5Ks (which were recently discovered) [14,15], more investigations on 5-IP7 have been performed than on 1-IP7 and IP8. Importantly, it should be noted that deletion or overexpression of IP6Ks could impact the level of both 5-IP7 and IP8. Thus, careful interpretations are needed to evaluate whether phenotypes obtained from pharmacological or genetic modifications in IP6K could reflect selective actions of each of the PP-IPs. A prime example is the recent discovery of the control of cellular phosphate efflux by IP8 but not 5-IP7 nor 1-IP7 [39,44]

### 4.1. Reproduction

Deletion of IP6K1 in mice leads to male sterility without, affecting the female reproductive system. Histological analyses show that IP6K1 knockout (KO) males have very few advanced spermatids in the seminiferous tubules and no sperm in the epididymis [45]. This indicates that IP6K1 plays a role in spermiogenesis, which is the final stage of spermatogenesis. Elongating IP6K1 KO spermatids are severely disoriented, and their acrosomes are degenerated during sperm differentiation [45]. In addition, defective IP6K1 KO sperm cells fail to release and are engulfed by surrounding Sertoli cells in the seminiferous epithelium of KO mice [46]. This induces male infertility in IP6K1 KO mice. IP6K1 is highly expressed in late-stage pachytene spermatocytes and in round spermatids within the mouse testis. IP6K1 in the round spermatids of mice is enriched in cytoplasmic granules, which are called chromatoid bodies [45]. These are ribonucleoprotein complexes involved in mRNA translational control, mRNA decay, and small RNA-mediated gene regulation. Deletion of IP6K1 results in disruption of the chromatoid body and premature synthesis of key proteins, such as transition nuclear protein 2 (TNP2) and protamine 2 (PRM2) [45]. These molecular defects in IP6K1 KO spermatids seem to drive abnormal nuclear DNA condensation in elongating spermatids. In addition, malformed KO spermatids undergo apoptosis [45]. Interestingly, normal male reproduction is found in IP6K2 and IP6K3 KO mice [47,48,49]; therefore, IP6K1 seems to be the key component of sperm differentiation. Further investigation is required to identify molecular targets that are directly modulated by the IP6K1 products 5-IP7 and IP8.

### 4.2. Neurological Effects

Exocytosis mediates important cellular functions by controlling the secretion of intracellular substances, such as hormones [50]. Therefore, this process is the key event for chemical communication between cells. In neurons, neurotransmitters are stored in synaptic vesicles. They are released into the synaptic cleft when a synaptic vesicle fuses with the cell membrane [51]. These processes are finely controlled by specialized proteins, such as soluble N-ethylmaleimide-sensitive factor attachment protein receptors (SNAREs) and synaptotagmin (Syt). When levels of calcium rise at the presynaptic terminal of neurons, calcium-bound Syt stimulates the docking and fusion of synaptic vesicles to the cellular membrane via highly controlled molecular interactions among presynaptic proteins, such as Syt and SNARE, and membrane lipids [50,51,52].

Elevating the expression of 5-IP7 in neuroendocrine PC12 cells via IP6K1 overexpression suppresses neurotransmitter release. In line with this, IP6K1 knockdown in PC12 cells decreases 5-IP7 and further promotes neurotransmitter release [37]. Furthermore, the depletion of IP6K1 in cultured hippocampal neurons increases action potential (AP)-driven synaptic vesicle exocytosis at synapses [37]. Recently, Park et al. have reported that hippocampal neurons from IP6K1 KO mice exhibit an increased presynaptic release probability [53]. Collectively, these findings suggest that 5-IP7 is a physiologic inhibitor of synaptic vesicle exocytosis.

Lee et al. have used biophysical analyses of reconstituted synaptic vesicles to reveal that 5-IP7 is the more potent at suppressing synaptic membrane fusion when compared with other IPs, such as IP6 and 1-IP7 [37]. Mechanistically, the molecular target for 5-IP7 is Syt1. 5-IP7 directly binds to the polybasic C2B domain of Syt1 with an affinity greater than one order of magnitude higher than that of IP6 [37]. Calcium and 5-IP7 do not compete with each other for Syt1 [37]; therefore, 5-IP7 can lock up Syt1 in a functionally inactive status.

Synaptic vesicle endocytosis determines neurotransmitter release by driving the invagination of the presynaptic plasma membrane to generate membrane-bound synaptic vesicles [54]. This synaptic vesicle cycling process enables neuronal recycling of the membrane for continuous synaptic vesicle cycling [54]. Interestingly, IP6K1 KO hippocampal neurons fail to supply synaptic vesicles to the recycling pool of synaptic vesicles following intensive neuronal stimulation [53]. This suggests that synaptic vesicles cannot be fully restored after synaptic vesicle exhaustion following the loss of IP6K1. Moreover, IP6K1 KO hippocampal neurons do not respond to dynasore, a selective pharmacological dynamin inhibitor, indicating that synaptic vesicle endocytosis is markedly impaired in IP6K1 KO neurons [53]. The exact targets for 5-IP7 in regulating synaptic endocytosis have not been defined. Therefore, further elucidation of the role of IP6K1 and 5-IP7 in controlling endocytic events is required.

Numerous studies have reported altered behavioral responses that are associated with IP6K1 deletion in mice [55]. IP6K1 KO mice exhibit responses in amphetamine-induced locomotion when compared with control mice [55]. In addition, social behavior is affected in IP6K1 KO mice; they exhibit autistic social behavior, such as a failure to explore the newly introduced mouse over a familiar animal [55]. This is indicative of decreased social motivation. Furthermore, IP6K1 KO mice exhibit fewer social events [55]. Similar behavioral phenotypes are observed when glycogen synthase kinase 3 (GSK3) is deleted in mice [56]. The IP6K1 product 5-IP7 can inhibit Akt kinase [25], and IP6K1 non-catalytically binds to GSK, to stimulate its activity [55]. Thus, IP6K1 deletion leads to reduced GSK3 activity in the brain [55], mediating the GSK3-dependent neural signaling pathways involved in behavior and psychiatric diseases. Further investigations into the impact of IP6K1 and 5-IP7 on the control of other behaviors, including learning and memory, may yield interesting results.

IP6K1 KO mice exhibit impaired locomotion without unaffected motor coordination under basal conditions [55]; however, the locomotion of IP6K2 KO mice is impaired locomotive, including a reduced latency to fall off the rotarod, notable decrease in stride length, and speed of movement [57]. This indicates defective motor coordination. IP6K2 determines the nuclear translocation of 4.1N, a high-affinity IP6K2-binding protein [57], which is the key for its functions. Both IP6K2 and 4.1N are highly enriched in cerebellar granule cells. Their interaction controls the morphology of Purkinje cells and cerebellar synapses [57]. Therefore, the contribution of 5-IP7 to the cerebellar control by IP6K2 warrants further investigation.

IP6K3 is highly expressed in the cerebellum. IP6K3 KO mice exhibit aberrant motor learning, whereas overall activity in an open field is normal; however, additional gait analysis shows overlapped hind- and forepaw patterns [49]. These phenotypes are observed in mice with Purkinje cell dysfunction [58]. This indicates that interactions between IP6K3 and the cytoskeletal proteins adducin and spectrin leads to impaired synaptic structure and number in the Purkinje cells of IP6K3 KO mice [49].

### 4.3. Metabolic Homeostasis

The impact of 5-IP7 on energy homeostasis was revealed by genetic deletion of IP6K1 in mice. IP6K1 KO mice are smaller than wild-type mice, exhibiting a 15%–20% weight reduction from young-to-adult development [25]. Interestingly, IP6K1 KO mice exhibit normal food intake, indicating that this growth defect is independent of feeding control [25]. Levels of circulating insulin are lower in IP6K1 KO mice, reflecting the role of 5-IP7 in the promotion of pancreatic beta cells; a key feature of IP6K1 KO mice is insulin hypersensitivity [25]. Upon cellular activation by insulin, 5-IP7 production by IP6K1 is enhanced. Mechanistically, 5-IP7 is a negative feedback signal that inhibits Akt kinase, which is a key mediator of insulin action. Moreover, 5-IP7 acts as a competitive inhibitor to interfere with the interaction between the PH domain of Akt and phosphatidylinositol-3,4,5-trisphosphate [25]. This suppresses the translocation of cytosolic Akt to the plasma membrane. Therefore, deleting IP6K1 and its product 5-IP7 leads to the hyperactivation of Akt and downstream insulin signaling events, such as protein synthesis. In line with this, high-fat-diet-fed IP6K1 KO mice are protected from obesity, hyperinsulinemia, hyperglycemia, hepatic steatosis, and insulin resistance [25]. Increased Akt in IP6K1 KO mice stimulates fatty acid oxidation and reduces the accumulation of fat via GSK3ß inhibition, which promotes adipogenesis [25]. Furthermore, IP6K1 deletion in mice ameliorates aging-induced obesity and insulin resistance [25]. Interestingly, increased energy expenditure is the key to the metabolic benefits found in whole-body IP6K1 KO mice.

Chakraborty et al. have identified the underlying mechanism associated with the lean phenotype of IP6K1 KO mice via adipocyte-specific IP6K1 KO mice [59,60]. Adipose tissue is a central metabolic organ in the regulation of energy homeostasis. White adipose tissue stores extra energy, and brown adipose tissue mediates cold-induced adaptive thermogenesis [61,62]. When IP6K1 is deleted in adipocytes, mice exhibit enhanced thermogenic energy expenditure, which is protective against high-fat-diet-induced obesity at ambient, but not thermoneutral, temperatures [59,60]. The mechanistic basis of this action is associated with AMP-dependent protein kinase (AMPK), which is a sensor for cellular energy. When cellular ATP depletes and the AMP/ATP ratio rises, AMPK becomes activated, which potently stimulates metabolic activity of brown and beige fat to maintain metabolic homeostasis, including body weight and insulin sensitivity [63]. IP6K1 has been reported to be antagonistic to AMPK; IP6K1 KO promotes AMPK-dependent energy expenditure. For example, IP6K1 deletion in adipocytes leads to increased cold-induced beige adipogenesis and transformation of white adipocytes into brown-like thermogenic beige cells when compared with wild-type mice [59].

Interestingly, local IP6 activity is the key to IP6K1 action in the control of AMPK. IP6, a substrate of IP6K1, stimulates upstream kinase-mediated phosphorylation of the catalytic subunit of AMPK [59]. When IP6 is converted to 5-IP7 or IP5 by IP6K1, IP6K1 loses the action of IP6 in activating AMPK [59]. In addition, pharmacological inhibition of IP6K by the IP6K inhibitor TNP [N2-(m-trifluorobenzyl), N6-(p-nitrobenzyl)purine] increases AMPK phosphorylation and AMPK signaling events in the adipocytes of high-fat-diet-fed mice [59]. More detailed perspectives on the IP6K-mediated control of metabolic homeostasis by IP6Ks have been reviewed [64].

Cellular IP8 levels responded to cellular energetic stress [65]; the levels of IP7 were not affected. A recent study has reported that PPIP5Ks play a role in the growth and metabolism of cancer cells [66]. Double deletion of both PPIP5K1 and PPIP5K2 depletes IP8 and increases 5-IP7 in HCT116 colon cancer cells. These PPIP5K KO cells show reduced cellular growth and increased ATP expression [66]. Furthermore, glycolysis and mitochondrial mass/activity are increased in these PPIP5K KO cells, indicating that PPIP5Ks and PP-IPs play a role in cellular metabolism [66]. Additional data are required through the use of animal models, to elucidate the mechanism of action of PPIP5Ks and their physiological impact in cancer cell growth.

### 4.4. Aging

IP6K3 KO mice have an extended lifespan when compared with wild-type mice [48]. In addition, they exhibit a lower body weight, blood glucose level, and circulating insulin. IP6K3 KO mice have a reduced fat mass and exhibit improved glucose tolerance, which suggests that IP6K3 deletion promotes enhanced metabolic functions [48]. IP6K3 expression is increased in muscle cells in response to dexamethasone treatment or metabolic stresses, such as diabetic, fasting, and muscle denervation [48]. Furthermore, IP6K3 deletion reduces the phosphorylation of the S6 ribosomal protein in the heart [48]. Further studies are required to elucidate the molecular links between IP6K3, PP-IPs, and KO phenotypes. In addition, the IP6K isoform-specific contribution to lifespan control has not been reported.

### 4.5. Blood Clotting

Ghosh et al. first reported delayed platelet aggregation times in IP6K1 KO mice that exhibited increased blood-clotting times. IP6K1 KO mice show longer bleeding times, which is protective against pulmonary thromboembolism [67]. One major factor of controlling platelet aggregation is the level of polyphosphates in platelets. Budding yeast experiments have shown that PP-IP metabolism and 5-IP7 are critical for the maintenance of polyphosphate levels [67]. IP6K1 KO platelets contain reduced polyphosphate levels, which leads to impaired blood clotting [67]. In addition, polyphosphates stimulate neutrophil-platelet aggregation (NPA), which is critical in the promotion of neutrophil accumulation in alveolar spaces in lung inflammation [68]. The reduced polyphosphate production in IP6K1 KO platelets results in decreased NPA formation, which lowers neutrophil accumulation. Therefore, IP6K1 deletion in platelets alleviates the lung damage caused by bacterial pneumonia [68]. Taken together, these studies reveal that 5-IP7 is essential for platelet functioning by indirectly modulating polyphosphate levels. This novel role for 5-IP7 should be further defined at molecular levels.

### 4.6. Keratoconus

Keratoconus is a disorder of the eye in which progressive thinning of the cornea and its outward conical protrusion occur, resulting in blurry vision, nearsightedness, astigmatism, and light sensitivity. Recent whole-genome sequencing in familial keratoconus patients identified mutations in PPIP5K2 that were responsible for 1-IP7/IP8 synthesis [69]. PPIP5K is unique because it contains both kinase and phosphatase domains, which are the biochemical foundations of the futile cycle [27]. The N843S PPIP5K2 mutation within the phosphatase domain leads to a reduction in phosphatase activity, which suggests increased kinase action [69]. A gene-trap mouse model harboring a small deletion of the PPIP5K2 phosphatase domain leads to irregularities on the surface of the cornea and pathological corneal thinning, which are similar to keratoconus [69]. However, the exact role of 1-IP7 and IP8 in the control of keratoconus should be further defined.

### 4.7. Hearing

Genetically complex non-syndromic recessively inherited hearing loss (NSRHL) accounts for approximately 75% of hereditary deafness and is a genetically heterogeneous disorder [70]. Analyses of two large consanguineous Pakistani families segregating NSRHL have revealed a missense mutation (R837H) in the phosphatase domain of PPIP5K2 [71]. The R837H variant reduces the phosphatase activity of PPIP5K2, which elevates its kinase activity [71]. Mice homozygous for a targeted small deletion of the PPIP5K2 phosphatase domain show degeneration in cochlear outer hair cells with elevated hearing thresholds [71]. This supports a role for PPIP5K2 in hearing control; however, the molecular targets for 1-IP7 and IP8 and signaling actions should be further elucidated.

### 4.8. Cancer

The regulation of cellular migration is mediated by IP6K1. When IP6K1 is depleted in mouse embryonic fibroblasts and cancer cells, such as HeLa and HCT116, they undergo morphological changes that are associated with decreased cell migration [72,73,74]. Moreover, 5-IP7 deletion is associated with a reduction in tyrosine phosphorylation and focal adhesion kinase (FAK) activity [73]. In addition, IP6K1-depleted HCT116 colorectal cancer cell xenografts in immunocompromised mice exhibit reduced invasion when compared with wild-type mice [72]. Moreover, IP6K1 deletion in mice protects against 4-nitroquinoline-1-oxide (4-NQO)‒induced carcinogenesis and diminishes the progression of invasive carcinoma [74]. Taken together, these data indicate that IP6K1 is a physiological determinant of cancer cell migration.

IP6K1 and 5-IP7 have been reported to control the DNA damage response in nucleotide excision repair. Under basal conditions, IP6K1 forms a ternary complex with Cop9 de-ubiquitination signalosome (CSN) and Cullin. Furthermore, 5-IP7 dissociates the CSN–Cullin complex in response to ultraviolet light, which activates Cullin-dependent nucleotide excision repair [75]. Furthermore, IP6K1 KO mice do not spontaneously develop tumors [75]. These data are critical for targeting IP6K1 in vivo because IP6K1 inactivation can stimulate Akt kinase in insulin-stimulated metabolic control [25,76]. Therefore, further research is required to fully explore the impact of IP6K1 on the cellular characteristics of cancer in vivo.

IP6K2 acts to control p53 catalytically. Depletion of IP6K2 in various cancer cells (OVCAR3, HeLa, and HL60) protects cellular apoptosis in response to cisplatin, etoposide, and other various cytotoxic reagents [77]. IP6K2 KO mice show improved survival following irradiation [47], suggesting that IP6K2 is a key factor in sensitizing cancer cell death. At the molecular level, IP6K2-mediated 5-IP7 acts to determine the function of p53; it binds to p53 and inhibits the p53-mediated induction of cell-cycle-arrest genes, such as p21. As such, IP6K2 stimulates p53 apoptotic regulators, such as Noxa [77]. The activation of p53 is mediated by phosphoinositide-3-kinase-related kinases (PIKKs), DNA dependent protein kinase (DNA-PK), and ataxia–telangiectasia-mutated protein kinase (ATM). PIKKs are stabilized by the TTT complex [78]. Mechanistically, 5-IP7 binds and activates casein kinase 2 (CK2) and promotes the phosphorylation of the TTT complex, which stimulates DNA-PK/ATM to activate p53 [36]. Another target for IP6K2 is TRAF2. IP6K2 non-catalytically interacts with TRAF2, which attenuates transforming growth factor beta-activated kinase-1 (TAK1) phosphorylation and the activation of NF-κB to trigger cell death [79]. Therefore, TNF-mediated apoptotic programming can be blocked by IP6K2 deletion.

IP6K2 can affect cell–matrix adhesion and lower cell–cell adhesion. IP6K2 KO HCT116 colorectal cancer cells show growth retardation with reduced cell–matrix adhesion; however, cell–cell adhesion is strengthened [72]. Xenograft tumors from IP6K2 KO HCT116 cells exhibit reduced tumor growth and lower metastatic potential [72]. IP6K2 directly binds to the tumor suppressor, LKB1, which inhibits cell migration via cytosolic tyrosine phosphatase activation [72]. Interestingly, LKB1 cannot be properly sequestered in the nucleus in the absence of IP6K2, which increases tyrosine phosphatase activity and stimulates the dephosphorylation of focal adhesion kinase (FAK) and related cell migration [72]. Therefore, IP6K1-mediated control of FAK phosphorylation and IP6K2-dependent LKB-FAK signaling should be further investigated to define the role of 5-IP7.

IP6K2 KO mice do not exhibit defects in development and metabolic homeostasis, or spontaneous tumors [47]. However, IP6K2 deletion in mice promotes the formation of aerodigestive tract carcinoma in response to carcinogen 4-NQO [47]. Using the 4-NQO-mediated chemical carcinogenesis model, studies have shown that IP6K2 and IP6K1 KO mice have accelerated and attenuated tumorigenesis, respectively [47,74]. This may reflect the IP6K2-specific control of p53. Future studies are required to extricate the complexity of IP6K isoform-specific roles in cancer. 

In addition to PP-IP−mediated biological functions introduced above, other latest findings on PP-IP actions are listed in Table 1.

## 5. Conclusions and Perspectives

**Figure 3:**
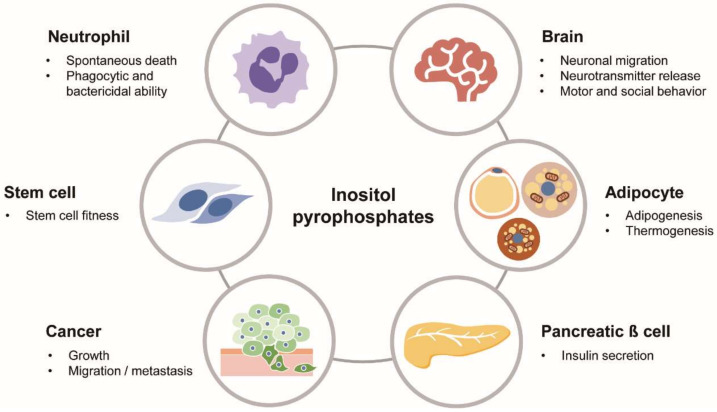
Biological roles of PP-IPs in physiology and diseases. PP-IPs as versatile signaling molecules play various biological actions in different cells/organs and pathological conditions, including cancer.

PP-IPs are signaling molecules evolved to mediate various biological actions (Figure 3). Although our knowledge of PP-IPs has been substantially expanded, many questions about their activities remain open. Identifying direct protein targets for PP-IPs will be the most challenging work. Some protein targets may discriminate among IP7 metabolites. For example, the binding affinity of 5-IP7 for Syt1 is much higher compared to the Syt1‒1-IP7 interaction, indicating that the selective binding of 5-IP7 to Syt1 binding does not simply reflect the number of negative charges on IP7 but, rather, the specific structural features of PP-IPs [37]. To describe the endogenous PP-IP‒protein interactome, advanced screening strategies are needed.

The development of animal models for cell-type-specific deletions in IP6Ks and PPIP5Ks will expand our understanding of the in vivo roles of PP-IPs. The in-parallel development of pharmacological reagents is also needed to investigate effects on acute inhibition of IP6Ks, PPIP5Ks, or other upstream inositol polyphosphate kinases, such as IPMK [91,92,93]. Recently, Shears and colleagues discovered quercetin and its derivative as IP6K-specific inhibitors [92]. Since IP kinases can play non-catalytic roles, selective suppression of catalytic activities of IP6Ks and PPIP5Ks will allow us to focus on their PP-IP-dependent signaling actions. In addition to typical ways to modulate cellular PP-IPs by overexpressing IP6Ks or PPIP5Ks in mammalian cells, it is possible to directly deliver photocaged PP-IPs into cells [84]. Th development of cell-permeable, regulated PP-IP reagents will be useful to validate biochemical and cellular changes led by PP-IPs.

Recent reports have unveiled genetic variations of IP6Ks and PPIP5Ks in diseases [4] such as Alzheimer’s disease [94], hearing loss [71], and keratoconus [69]. Establishing a tight link between PP-IPs and clinical findings will be important to translate scientific knowledge into clinical diagnostic and therapeutic applications. Mass spectrometric measurement of PP-IPs was recently attempted [95,96], applying a highly sensitive platform to the detection of PP-IPs. We strongly believe that advanced detection techniques and a comprehensive understanding of the roles of PP-IPs in cellular signaling networks will lead to the development of valuable tools to manage leading human diseases, like metabolic syndromes and cancer.

## Figures and Tables

**Table 1:** Physiologic functions of inositol pyrophosphates.

Functions	PP-IPs	Biological Models	Phenotypes	Reference
Type I IFN-mediated viral immunity	1-IP7	PPIP5K1/2 KD HEK293	Decreased cellular type 1 interferon immune response	[38]
Cell growth and metabolism	5-IP7/IP8	PPIP5K1/2 DKO HCT116 colorectal cancer cells	Hypermetabolic, growth inhibition	[66]
Prelingual sensorineural deafness	IP8	Phosphatase domain-deleted PPIP5K2 knock-in mice	High-frequency progressive hearing loss	[71]
Keratoconus	IP8	Phosphatase domain-deleted PPIP5K2 knock-in mice	Corneal pathological phenotypes	[69]
Cellular phosphate homeostasis	IP8	HCT116 Saos-2	Dynamic turnover of IP8 by P_i_ Reduced Pi efflux and accelerated differentiation into a biomineralization	[27,39]
	5-IP7/IP8	IP6K1/2 DKO HCT116	Decreased phosphate import/export Increased cellular ATP and phosphates	[44]
Growth factor signaling cascade	5-IP7/IP6	PPIP5K1 KD L6 Myoblasts	Reduced SIN-mediated mTORC2 activation	[80]
Energy dynamics	5-IP7	IP6K1 KO MEFs	Increased ATP and reduced mitochondrial respiration	[41]
Glucose homeostasis and insulin sensitivity	5-IP7	IP6K1 KO mice	Resistant to obesity and diabetes (HFD and age)	[25]
		Prediabetic patients and C2C12 myotubes	High intensity exercise reduced muscle IP6K1 and improved insulin sensitivity	[81]
		Young adult obese patients and C2C12 myotubes	Increased muscle IP6K1 after lean meat ingestion in obese group	[82]
Adipose tissue metabolism	5-IP7	IP6K1 KO mice	Increased fat breakdown and impaired adipogenesis	[25,76]
	IP6/5-IP7	IP6K1 KO or AdKO mice	Increased thermogenic activity	[59,60,76]
Beta cell insulin secretion	5-IP7	IP6K1 KD mouse beta cell (MIN6)	5-IP7 triggers insulin exocytosis via regulating Ca^2+^ oscillation Glucose induces increase of 5-IP7 in beta cells	[21,83,84]
Neurotransmitter release	5-IP7	IP6K1 KD hippocampal neurons and PC12	5-IP7 suppresses synaptic vesicle exocytosis	[37]
		IP6K1 KO mice	Increased excitatory synaptic vesicle release (impaired synaptic endocytosis)	[53]
Viral particle exocytosis	5-IP7	IP6K1 OE HeLa	Attenuated release of HIV-1 virus-like particles	[40,42]
Vesicle trafficking	5-IP7	IP6K1 KO MEFs	Impaired dynein-driven transport	[43]
Chromatin remodeling	5-IP7	IP6K1 KO MEFs and KD HEK293T	Increased JMJD2C-dependent H3K9me3 demethylation	[85]
DNA damage and repair	5-IP7	IP6K1 KO MEFs IP6K1 KD HCT116	Promoted UV-induced NER and apoptosis	[75]
Neutrophil activity	5-IP7	IP6K1 KO mice	Enhanced bacterial killing (phagocytosis)	[86]
			Augmented nicotine-induced lung inflammation (delayed spontaneous death)	[87]
Spermatogenesis	Unknown	IP6K1 KO mice	Defective germ cell differentiation and development	[45,46]
Neuronal migration	5-IP7	IP6K1 KO MEFs	Neuronal migration defects and brain malformation	[73]
		IP6K3 KO mice		[88]
Cancer	5-IP7	IP6K1 KD HeLa and HCT 116 IP6K1 KO mice	Reduced migration/invasion and anchorage-independent growth Resistant to 4-NQO induced carcinogenesis	[74]
		IP6K2 KO HCT116	Inhibited tumor growth and metastasis	[72]
		IP6K2 KO mice	Susceptible to carcinogen (4-NQO) induced carcinogenesis	[47]
Behavior and brain function	5-IP7 (partial)	IP6K1 KO mice	Disruptive locomotor activity and social behavior	[55]
	Unknown	IP6K2 KO mice	Disrupted cerebellar disposition and psychomotor behavior	[57]
Stem cell fitness	5-IP7	IP6K1 KO BM-MSC	Increased growth and survival. Enhanced osteogenic potential	[89]
Apoptosis	5-IP7	IP6K2 KO HCT116	Resistant to genotoxic stress (apoptotic cell death)	[36,77]
Autophagy	5-IP7	BS-MSCs	TNP decreased hypoxia-induced autophagy	[90]
Aging and metabolism	Unknown	IP6K3 KO mice	Resistant to age-induced obesity and diabetes Extended lifespan	[48]

JMJD2C, jumonji domain 2C; NET, neutrophil extracellular traps; OE, overexpression; KO, knockout; KD, knockdown; DKO, double-knockout.
